# Fabrication of Titanium Nitride Thin Film on Titanium Using Cathodic Arc Plasma Evaporation for Biomedical Application

**DOI:** 10.1055/s-0045-1810016

**Published:** 2025-07-23

**Authors:** Trung Van Trinh, Hung Thai Le, Bang Le Thi

**Affiliations:** 1 School of Materials Science and Engineering, Hanoi University of Science and Technology, No.1 Dai Co Viet, Hanoi, Vietnam

**Keywords:** biomedical application, cathodic arc plasma deposition, titanium, titanium nitride, thin film

## Abstract

**Objectives:**

Commercially pure titanium (Cp-Ti) is often used for biomedical implant devices but has low hardness and wear resistance; therefore, it is not suitable for use in the sliding parts or joints. Owing to their good wear resistance and biocompatibility, titanium nitride (TiN) coatings are used to improve these surface properties of Ti. This study aims to fabricate TiN on Cp-Ti by cathodic arc plasma evaporation and to investigate the effect of Cp-Ti substrate temperature on the properties of coated TiN thin films for biomedical applications.

**Materials and Methods:**

Coated TiN thin films were deposited on Cp-Ti at different substrate temperatures of 25, 100, 175, and 250°C. The surface morphology, roughness, phase composition, hardness, coating adhesion, and biocompatibility of the TiN coatings were investigated using a digital optical microscope, scanning electron microscope, X-ray diffractometer, hardness tester, and *in vitro* cell studies.

**Statistical Analysis:**

Statistical differences were evaluated using analysis of variance (ANOVA) and Tukey's multiple comparison analysis, with statistical significance set at *p* < 0.05.

**Results:**

Thin films with a primary TiN phase were formed on the surface of the Cp-Ti substrate regardless of substrate temperatures. There was no significant difference in surface hardness between the coated samples even though the sample coated at 100 and 175°C showed a slightly higher values, ranging from 193 to 199 HV. Interestingly, surface roughness and coating adhesion were significantly influenced by substrate temperature. The higher the substrate temperature, the greater the surface roughness, while the best adhesion, with the hardness of 176 HV, was obtained at substrate temperature of 25°C. *In vitro* cell study indicated that the baby hamster kidney cells on the coating surface have grown and proliferated better than those on the uncoated surface.

**Conclusions:**

The TiN thin film was successfully coated on Ti by cathodic arc plasma evaporation at different substrate temperatures, ranging from 25 to 250°C. The adhesion of the coating at low substrate temperature (25°C) was the best compared to other substrate temperatures of 100, 175, and 250°C. *In vitro* cell studies have demonstrated the biocompatibility of the coated TiN thin film.

## Introduction

Commercially pure titanium (Cp-Ti) and Ti alloys, particularly Ti-6Al-4V, are widely used in dental and orthopaedic applications. Ti alloys exhibit superior strength and fatigue resistance, and a lower elastic modulus compared to pure Ti, making them more suitable for most load-bearing biomedical implants. However, the Ti-6Al-4V alloy may release Al and V ions, which have been associated with biological concerns, that is, cytotoxic and allergic reaction. 1 On the other hand, Cp-Ti is widely considered the best biocompatible metallic material, owing to its surface properties, which allow the spontaneous formation of a stable and inert oxide layer. Therefore, Cp-Ti is referred for use in places where corrosion resistance and biocompatibility are prioritized over mechanical strength. In addition, studies have shown that the osseointegration and biomechanical anchorage of Cp-Ti and Ti-6Al-4V are comparable. 2 However, Cp-Ti has relatively low hardness and wear resistance, thus hindering its application in joints where the sliding surface is required.

Titanium nitride (TiN) is well known for its light weight, high hardness, excellent wear resistance, high corrosion resistance, nontoxicity, and high biocompatibility. Thus, TiN is an ideal medical material suitable for coating on implants and surgical instruments, such as orthodontic devices, orthopaedic implants, hip prostheses, and cardiovascular biomaterials. 3 4 5 6 Moreover, the Food and Drug Administration recognizes TiN as a desirable material for blood contact applications. 3 Therefore, coating Ti with TiN thin films could be a potential approach to increase the surface properties of Ti in terms of hardness and wear resistance.

TiN coatings on Ti have been carried out by various coating techniques, including sputtering, pulsed laser deposition, and cathodic arc plasma evaporation (CAPE). Among them, CAPE has become especially prominent due to its ability to produce coatings with strong adhesion and high deposition rates. 7 8

In the CAPE method, substrate temperature is one of the most important technological parameters because it affects the characteristics of the coating, and the structure and properties of the substrate. High substrate temperatures (above 150°C) result in dense, crystalline coatings; however, substrate softening may occur at these high temperatures. As a result, CAPE is mostly conducted at remarkably low temperatures with a substrate temperature-to-melting temperature (T _s_ /T _m_ ) ratio of 0.1–0.3. 9

Although low-temperature CAPE processing is of interest, most previous studies were conducted on TiN coatings at relatively high temperatures (above 150°C) and focused on steel substrates, 10 11 while the influence of substrate temperature on the coating deposited in the low-temperature range (≤250°C), including room temperature (25°C), has not been systematically studied. For example, Vieira and Nono 11 characterized TiN thin films deposited by CAPE on AISI D6 tool steel at 220 and 450°C and found that the thickness of the TiN films increased with substrate temperature. In another study, the TiN coatings were deposited on high-speed steel at substrate temperatures of 150, 450, 10 and 400°C. 12 The results showed that the coating at a low temperature (150°C) exhibited a lower friction coefficient and surface roughness than that deposited at a high temperature (450°C). 10 Similarly, M2 steel was coated with TiN at a substrate temperature of 350°C. 12 However, coating adhesion, hardness, and roughness were not investigated.

The present study investigated the influence of substrate temperature (25, 100, 175, and 250°C) on the properties of TiN coatings deposited on a Cp-Ti substrate by CAPE. In addition, the coating properties such as coating adhesion, hardness, and roughness were also evaluated. Furthermore, *in vitro* cell study using baby hamster kidney cells was also conducted to evaluate the biocompatibility of the coatings for biomedical applications.

## Materials and Methods

In this study, TiN was coated on Cp-Ti substrate by CAPE. The deposition parameters of CAPE, particularly arc current, 7 substrate bias, 13 nitrogen partial pressure, 14 time, 15 16 and temperature, 11 are crucial in determining the properties and performance of the resulting coatings. Optimal deposition parameters, including arc voltage, arc current, Ar/N _2_ gas flow rate ratio, substrate rotation, and deposition times for the Ti interlayer and coating, were established through preliminary experiments. Detailed process parameters for the TiN coating are provided in Table 1 .

**Table 1:** Detail process parameters for cathodic arc plasma evaporation (CAPE) of titanium nitride coatings

Samples Parameters	M1	M2	M3	M4
Substrate temperature (°C)	25	100	175	250
Arc voltage (V)	18			
Arc current (A)	50			
Deposition time for Ti interlayer (min)	5			
Deposition time for coating (min)	10			
Gas flow rate ratio of Ar/N _2_ (mL/mL)	10/100			
Substrate rotation (rpm)	5			

The chemical composition of the Cp-Ti substrate is shown in Table 2 . The samples were cut into to a size of Ø15 mm × 2 mm. Deposition was carried out using a CAPE system (DADA, DSA-3000).

**Table 2:** Chemical composition of the commercially pure titanium (Cp-Ti) substrate

Cp-Ti	Al	V	Si	Ni	C	Cr	Ti
Chemical composition, wt.%	0.004	0.008	0.053	0.003	0.019	0.026	Balance

All samples were mechanically polished using SiC papers with grits of 400, 600, 800, and 1,200, followed by polishing with 1-µm alumina suspension. They were then ultrasonically cleaned in acetone and deionized water, air-dried, and placed in the CAPE chamber. A turbomolecular pump evacuated the chamber to a pressure of 1 × 10 ^−6^ torr. Ti (99.95%) served as the coating material, and nitrogen gas (99.99%) was used as the reactive gas. A titanium interlayer thin film of approximately 0.25 µm thickness was initially deposited on the polished Cp-Ti substrate surfaces for adhesion improvement of the TiN coating films. 11 17 18

The TiN films were then deposited at substrate temperatures of 25, 100, 175, and 250°C. Several pieces of single-crystal silicon wafer were coated under the same conditions as the study samples for cross-sectional scanning electron microscopy (SEM) imaging.

The morphology and surface roughness (S _a_ ) of the samples were examined using a digital optical microscope (Keyence, VHX-7000). The phase composition of the coatings was analyzed using an X-ray diffractometer (Panalytical, AERIS). The structure and chemical composition of the coatings were analyzed by SEM coupled with energy dispersive spectroscopy (EDS; JEOL, JSM-IT200). Hardness was measured with a microhardness tester (Struers, Duramin-2), and adhesion of coating was assessed according to the VDI3198 standard (Daimler-Benz method). 19 20 The biocompatibility in terms of initial cell attachment and cell proliferation of uncoated (commercially pure Ti) and TiN-coated Ti samples was evaluated through an *in vitro* cell test using baby hamster kidney (BHK) cells. BHK cells were chosen due to the ease of culture, and they are one of the standard cell lines used for biocompatibility and the cytotoxicity tests according to ISO 10993-5:2009. The cells were cultured in a growth medium containing Dulbecco's modified eagle medium (Thermo Fisher Scientific), 1% antibiotic (Thermo Fisher Scientific), and 10% fetal bovine serum (Thermo Fisher Scientific) in a humidified atmosphere with 5% CO _2_ at 37°C. Prior to the cell study, the samples were sterilized in an autoclave at 120°C for 20 minutes and then transferred to a 24-well plate. For initial cell attachment, the cells at a density of 10,000 cells/mL were seeded on each specimen and cultured for 24 hours. The BHK cells on the samples were fixed with 4% paraformaldehyde in phosphate buffer saline (PBS; Sigma-Aldrich) for 10 minutes, washed in PBS, and hydrated with a series of ethanol from 50 to 100% for 30 minutes each at 4°C. The initial cell attachment and morphology were observed under SEM after coating with platinum. For cell proliferation, the BHK cells at a density of 10,000 cells/well were cultured on the samples and incubated for 3 and 5 days. After the predetermined period, the cells were washed with PBS and incubated in 10% cell counting kit 8 (WST-8/CCK-8; Dojindo) and growth medium for 2 hours. Finally, 200 μL of the final medium was measured for absorbance at 560-nm wavelength using an enzyme-linked immunosorbent assay (ELISA) microplate reader (Chromate 4300).

Statistical differences were evaluated using analysis of variance (ANOVA) and Tukey's multiple comparison analysis, with statistical significance set at *p* < 0.05.

## Results and Discussion

**Fig. 1:**
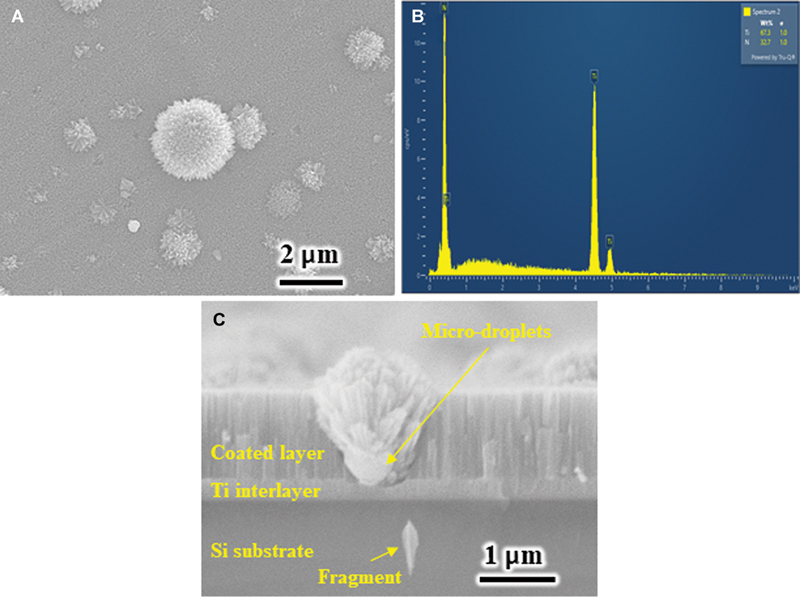
( **A** ) The top view scanning electron microscopy (SEM) image, ( **B** ) the EDS analysis of the M1 sample, and ( **C** ) the cross-sectional SEM image of a coated single-crystal silicon wafer, which was deposited together with the M1 sample.

The surface morphologies of the coatings were similar regardless of the substrate temperature. Fig. 1 presents representative SEM images of the top view and cross-section of coated sample and the results of EDS analysis of the M1 sample. Numerous cauliflower-shaped microdroplets (macroparticles) with different sizes were visible on the coating surface ( Fig. 1a ). The EDS result confirmed the chemical composition of the coated layer, which contained 67.3 wt% Ti and 32.7 wt% N ( Fig. 1b ). In the cross-sectional SEM image ( Fig. 1c ), the intermediate Ti interlayer was approximately 0.25 µm thick. The coated layer exhibited a column-like morphology. The top coated layer had a thickness of approximately 1.38 µm.

**Fig. 2:**
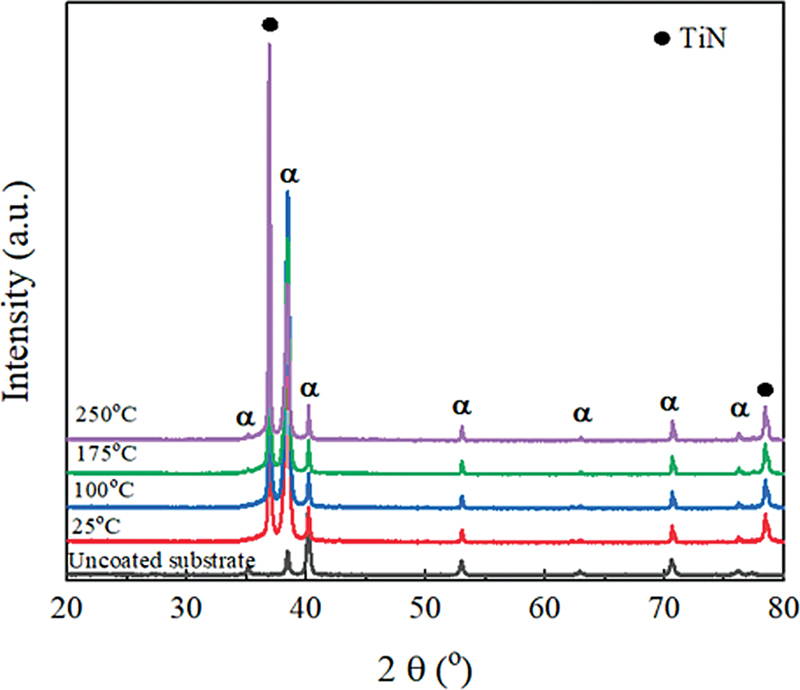
XRD patterns of the coated samples deposited at substrate temperatures of 25, 100, 175, and 250°C, as well as the uncoated substrate.

Fig. 2 illustrates the X-ray diffraction patterns of different samples. It is indicated that the coating primarily consists of the TiN phase (PDF-#87-0632) with preferred orientation in the (111) direction at 2θ = 36.8 degrees. The diffraction peaks of the Ti-α phase (PDF-#44-1294) were still observed in the XRD patterns of the coated samples. A possible reason for the detected α phase is that the coating is thin or the microdroplets have partially peeled off, generating craters, 9 which allow the X-ray diffraction signal to detect the Ti interlayer and/or the Ti substrate.

Table 3 shows the results of surface roughness analysis and hardness of the uncoated substrate and the coated samples at different substrate temperatures. The average surface roughness S _a_ of the uncoated substrate was ∼2.48 µm. All the coated samples had lower surface roughness (0.36–1.20 μm) than the uncoated substrate. The higher the temperature, the higher the surface roughness of the coating. This phenomenon may be due to the relatively strong agglomeration and growth of microdroplets and/or grains. 21 For titanium in orthopaedic applications, a positive influence on bone response to implant devices has been observed with roughness values ranging from 0.5 to 8.5 µm. 22 *In vitro* studies indicate that osteoblastic cell differentiation is optimal on micro-rough surfaces (∼3–4 µm) compared with that on smooth surfaces. By contrast, osteoclast activity is higher on smooth surfaces than on micro-rough ones. Hence, the ideal roughness for osseointegration is proposed to be 1–1.5 µm. 22 23 It is important to note that this proposed roughness value pertains to the substrate material. When dealing with coatings, additional factors like adhesion must be taken into account, as will be discussed later.

**Table 3:** Surface roughness analysis and hardness of the uncoated substrate and the coated samples at different substrate temperatures

Samples Parameters	Uncoated	M1	M2	M3	M4
Substrate temperature (°C)		25	100	175	250
Surface roughness (Sa; μm)	2.48	0.36	0.77	0.79	1.2
Surface hardness (HV _0.05_ ), *n* = 3	148 ± 4.0	176 ± 3.5	199 ± 3.9	193 ± 3.8	187 ± 3.7

The effect of substrate temperature on surface hardness of the coating was also evaluated, and the results are shown in Table 3 . The uncoated Ti sample exhibited a surface hardness of 148 HV. Coating Ti with TiN enhanced the hardness significantly. As the substrate temperature rose from 25 to 100°C, the surface hardness increased from 176 ± 3.5 to 199 ± 3.9 HV. However, the hardness decreased with further temperature increases to 175°C (193 ± 3.8 HV) and 250°C (187 ± 3.7 HV). It should be noted that the hardness value of the M1 samples was significantly different ( *p* < 0.05) with those of other samples. Although the hardness of the M2 sample was higher than that of the M3 and M4 samples, the difference was not statistically significant. This trend may be due to the pronounced oriented crystallization at elevated temperatures, leading to an increase in hardness. As the grain size or crystalline phase grew and the residual stress within the coating film decreased, the hardness subsequently declined. 9 16 Nevertheless, the variation in hardness among the coatings at different temperatures was not substantial. Patsalas et al 24 investigated the influence of substrate temperatures on the mechanical properties and structure of sputtered TiN thin films and revealed that temperatures (T _s_ ) ranging from 27 to 400°C have minimal impact on the film's mechanical properties.

**Fig. 3:**
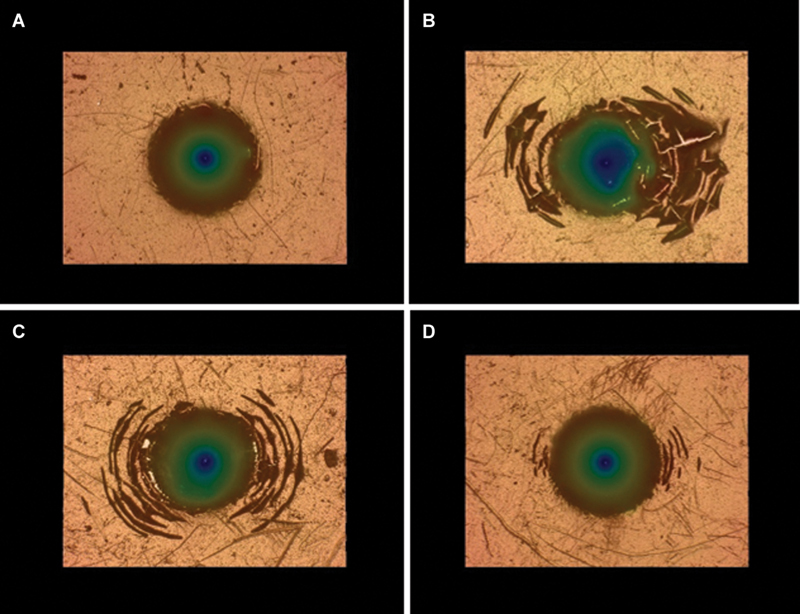
Imprints of the Rockwell C indentation on the surface of the coated samples at various substrate temperatures: ( **A** ) 25°C, ( **B** ) 100°C, ( **C** ) 175°C, and ( **D** ) 250°C.

Fig. 3 displays the imprints of the Rockwell C indentation on the surfaces of the coated samples at various substrate temperatures. These images were visually compared with HF1 (best adhesion) to HF6 (worst adhesion) in the VDI3198 standard to assess relative coating adhesion. The results indicate that sample M1 shows no peeling or cracking around the imprint, matching HF1 in the VDI3198 standard and thus demonstrating the best adhesion. Meanwhile, the other samples showed peeling and cracks around the imprint, indicating low adhesion levels. In particular, sample M2 exhibited the most extensive peeling, followed by sample M3. Sample M4 showed better adhesion than samples M2 and M3 but fell short of M1. On the basis of these findings and the hardness measurements, the adhesion strength of the coating is influenced by the hardness difference between the coating surface and the underlying substrate. Greater hardness differences tend to increase peeling, indicating a low adhesion. In biomedical implant applications, the strong adhesion between the coating and the substrate is preferred, 25 26 27 as it can prevent the formation of wear debris that may cause inflammatory response and implant failure. Therefore, sample M1 (coated at a low temperature of 25°C) with the best coating adhesion was selected for subsequent cell testing.

**Fig. 4:**
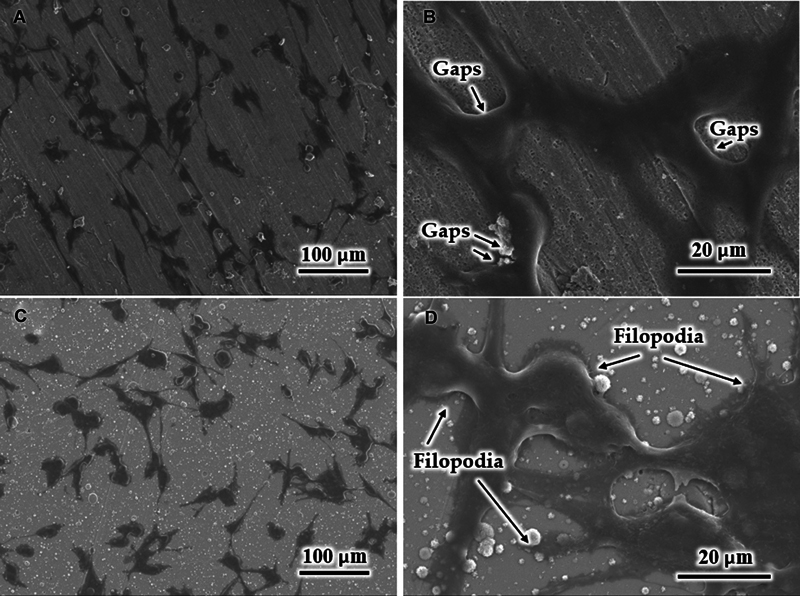
Scanning electron microscopy (SEM) images of the BHK cells on the surfaces of the ( **A, B** ) uncoated substrate and ( **C, D** ) coated M1 samples.

Fig. 4 shows the morphology of BHK cells after dehydration on the surfaces of the uncoated substrate and the coated M1 sample after 24 hours of culture. The cells were well attached to both surfaces with good spreading and occupied a large area ( Fig. 4a, c ). At high magnification, the lamellipodia of the BHK cells on the uncoated substrate ( Fig. 4b ) appeared short, and clear gaps were found between the cell matrix and the substrate. Meanwhile, the lamellipodia of the cells on the coated M1 substrate had penetrated and occupied the gaps ( Fig. 4d ). In addition, the filopodia visibly spread out and securely attached to the nanostructure of the coating, and the cauliflower-like particles were covered by the lamellipodia ( Fig. 4d ). This finding indicates the firm adhesion and migration of the cells on the coated sample.

**Fig. 5:**
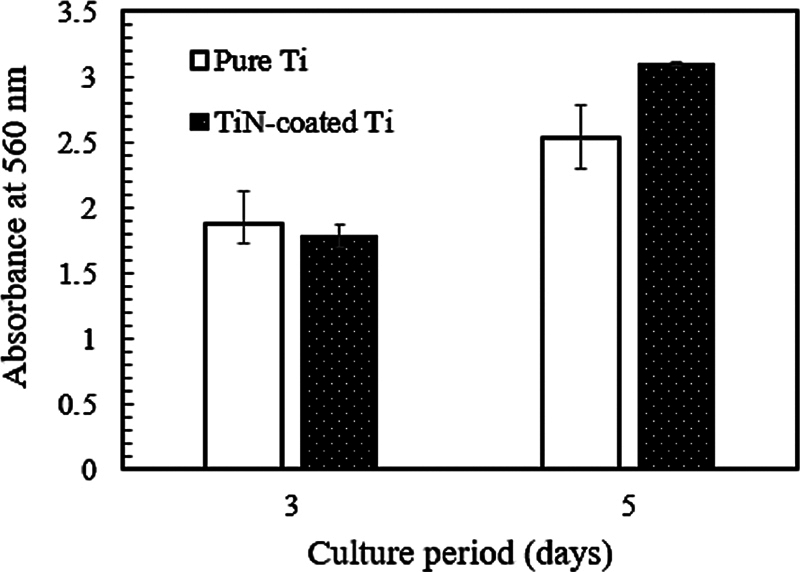
Cell proliferation on different titanium substrates.

Fig. 5 shows the cell proliferation on the surfaces of the uncoated substrate (pure Ti) and coated M1 sample (TiN-coated Ti) after being cultured for 3 and 5 days. In general, the cells have adhered to the two surfaces and proliferated during the culture period. On day 3, a higher absorbance was detected on the pure titanium surface, but the difference between the two surfaces was not statistically significant. After day 5, a higher absorbance was observed on the TiN-coated sample compared with that on the pure Ti. This finding demonstrated that the cells grown on the TiN-coated sample had a high proliferation rate. In line with the initial cell morphology, the significant spreading with filopodia and the firm adhesion of the cells' lamellipodia on the TiN-coated surface could enhance the proliferation of the cells.

These results indicate that TiN coating promotes the attachment and spreading of cells and subsequently their proliferation. The surface microstructure featuring a cauliflower-like texture and low roughness from TiN coating can promote protein adsorption and provide an excellent surface for cell adhesion, thus enhancing cell spreading and proliferation. 28 29

Protein adsorption is the first process that occurs after implantation of a biomaterial, followed by cell attachment, which are the prerequisites for further osseointegration. 30 Surface roughness of the implant is one of the main parameters influenced protein adsorption and, consequently, cell response. 31 32 33 34 A previous study had demonstrated that a surface roughness in the range of S _a_ = 0.48 to 0.77 µm enhances fibronectin absorption, which is crucial for subsequent cell attachment. 32 In addition, a roughness value of between 1 and 1.5 μm has been found to be most effective for bone integration. 33 In this study, it was found that S _a_ ranging from 0.36 to 1.2 μm could be favorable for cell attachment and proliferation, as demonstrated by the *in vitro* test, and may be a potentially support for improved osseointegration.

Although the BHK fibroblasts were used for the initial evaluation of biocompatibility, primarily to provide a general indication of the material's ability to support cell migration, attachment, and proliferation, other osteoblast cells or mesenchymal stem cell models provide a more accurate evaluation of bone–implant interaction. Further studies on bone–implant interaction could be conducted using osteoblast cells or mesenchymal stem cell models.

## Conclusion

This study investigated the influence of substrate temperature (25, 100, 175, and 250°C) on the properties of TiN coatings deposited on Cp-Ti substrate by CAPE. *In vitro* cell study using BHK cells was also conducted to evaluate the biocompatibility of the coatings for biomedical applications. Some main conclusions are summarized as follows:

- A TiN coating was deposited on the surface of Cp-Ti using CAPE, with a Ti interlayer added to enhance adhesion. The TiN coating also contained numerous macroparticles with a cauliflower-like shape.
- High substrate temperatures resulted in high surface roughness.
- The adhesion of the coating at low substrate temperature (25°C) was the best compared to other substrate temperatures of 100, 175, and 250°C.
- *In vitro* cell study indicated that the BHK cells on the coating surface have grown and proliferated better than those on the uncoated surface.
